# MR beyond diagnostics at the ESMRMB annual meeting: MR theranostics and intervention

**DOI:** 10.1007/s10334-024-01176-5

**Published:** 2024-06-12

**Authors:** Milan Hájek, Ulrich Flögel, Adriana A. S. Tavares, Lucia Nichelli, Aneurin Kennerley, Thomas Kahn, Jurgen J. Futterer, Aikaterini Firsiori, Holger Grüll, Nandita Saha, Felipe Couñago, Dogu Baran Aydogan, Maria Eugenia Caligiuri, Cornelius Faber, Laura C. Bell, Patrícia Figueiredo, Joan C. Vilanova, Francesco Santini, Ralf Mekle, Sonia Waiczies

**Affiliations:** 1 Department of Diagnostic and Interventional Radiology, Institute for Clinical and Experimental Medicine, Prague, Czech Republic; 2 Experimental Cardiovascular Imaging, Institute for Molecular Cardiology, Heinrich Heine University Düsseldorf, Düsseldorf, Germany; 3 Centre for Cardiovascular Sciences and Edinburgh Imaging, University of Edinburgh, Edinburgh, UK; 4 Sorbonne Université, Inserm, CNRS, UMR S 1127, Paris Brain Institute, ICM, Paris, France; 5 Department of Neuroradiology, AP-HP, Pitié-Salpêtrière Hospital, Paris, France; 6 Department of Sports and Exercise Science, Institute of Sport, Manchester Metropolitan University, Manchester, UK; 7 Department of Biology, University of York, York, UK; 8 Department of Diagnostic and Interventional Radiology, University of Leipzig, Leipzig, Germany; 9 Minimally Invasive Image-Guided Intervention Center (MAGIC), Department of Medical Imaging, Radboudumc, Nijmegen, The Netherlands; 10 Unit of Diagnostic and Interventional Neuroradiology, Diagnostic Department, University Hospitals of Geneva, Geneva, Switzerland; 11 Institute of Diagnostic and Interventional Radiology, Faculty of Medicine, University Hospital of Cologne, University of Cologne, Cologne, Germany; 12 Department of Chemistry, Faculty of Mathematics and Natural Sciences, University of Cologne, Cologne, Germany; 13 Max-Delbrück-Centrum Für Molekulare Medizin (MDC), Berlin Ultrahigh Field Facility, Berlin, Germany; 14 Charité-Universitätsmedizin Berlin, Berlin, Germany; 15 Experimental and Clinical Research Center (ECRC), A Joint Cooperation Between the Charité Medical Faculty and the MDC, Berlin, Germany; 16 Department of Radiation Oncology, Hospital Universitario San Francisco de Asís, Hospital Universitario Vithas La Milagrosa, GenesisCare, 28010 Madrid, Spain; 17 A.I. Virtanen Institute for Molecular Sciences, University of Eastern Finland, Kuopio, Finland; 18 Neuroscience Research Center, Department of Medical and Surgical Sciences, Università Degli Studi “Magna Graecia”, Catanzaro, Italy; 19 Translational Research Imaging Center (TRIC), Clinic of Radiology, University of Münster, Münster, Germany; 20 Early Clinical Development, Genentech Inc., South San Francisco, USA; 21 Institute for Systems and Robotics, ISR-Lisboa, Lisbon, Portugal; 22 Department of Bioengineering, Instituto Superior Técnico, Universidade de Lisboa, Lisbon, Portugal; 23 Department of Radiology, Clínica Girona, Institute of Diagnostic Imaging (IDI) Girona, University of Girona, 17004 Girona, Spain; 24 Department of Radiology, University Hospital of Basel, Basel, Switzerland; 25 Basel Muscle MRI, Department of Biomedical Engineering, University of Basel, Basel, Switzerland; 26 Center for Stroke Research Berlin (CSB), Charité - Universitätsmedizin Berlin, Berlin, Germany

## Introduction

The realm of MR intervention and theranostics is experiencing a rapid evolution. Within the broader scope of theranostics, paradigm shifts in non-invasive and invasive therapeutic intervention signify an era where treatment efficacy can be accurately tracked and assessed. The word Theranostics is a fusion of therapy and diagnostics, reflecting its dual purpose of treating and diagnosing diseases. It epitomizes a paradigm where treatment efficacy can be systematically monitored and optimized. Initially rooted in nuclear medicine with applications in diagnosing and treating cancer, it has undergone a transformative journey. Today, it encompasses a broader spectrum of imaging modalities, with MRI emerging as a non-invasive, patient-friendly, and potent clinical tool, alongside advancements in nanotechnology [1]. Far from being merely a trendy term, theranostics embodies a long-standing aspiration among scientists and clinicians to enhance patient care and advance personalized medicine. While some may view it as a buzzword [2] perhaps for securing research funding or driving healthcare policies, its essence lies in its potential to revolutionize healthcare delivery and outcomes. This evolution underscores the pivotal role of theranostics in pushing the boundaries of (molecular) imaging technologies to revolutionize patient care. Interventional MRI also has a rich history dating back to soon after the introduction of clinical diagnostic MRI [3]. Recognizing the superior soft tissue contrast capabilities of MRI, radiologists began exploring its use for guidance during interventional procedures, especially those involving head and neck lesions. Interventional MRI is a specialized domain where medical images do not only serve diagnostic purposes but also guide minimally invasive surgical or vascular procedures. Procedures including those involving small incisions in the body, are aimed at diagnosing, treating, and even curing various conditions. Today, it is widely employed to guide various invasive and noninvasive diagnostic and therapeutic interventions, such as robotic in-bore-targeted biopsies [4] and has immense theranostic potential, such as in deep brain stimulation [5].

As part of the program of the upcoming 2024 Annual Meeting of the European Society for Magnetic Resonance in Medicine and Biology (ESMRMB), the Congress Planning Committee has invited speakers from across Europe, experts in the field or MR theranostics and intervention, who will deliver plenary and educational talks in these corresponding fields.

## Content

The ESMRMB program divides the focus topic on MR theranostics and intervention into 4 main sessions: (1) Multimodal imaging for theranostics, (2) Invasive interventional MR, (3) Noninvasive interventional MR and (4) The role of MRI in drug development.

The plenary will delve into recent advances and prospects in theranostics, covering the synthesis, delivery, and application of new probes, designed with identifiable markers for precise drug localization within the body. Among preclinical molecular imaging methods, fluorine (^19^F) MRI has gained prominence [6], with background-free detection making fluorine-containing molecules ideal tracers for various MRI applications, including quantification of inflammatory disorders [7, 8] and treatment assessment [9, 10]. However, the low in vivo availability of administered fluorinated materials limits sensitive reporting. Nonetheless, innovations in ^19^F tracer design enable precise imaging of specific cell types [11] and measurement of physiologically important parameters like local oxygenation. Multi-targeted ^19^F nanotracers, equipped with binding molecules targeting specific immune cell subtypes, enable comprehensive mapping of immune response dynamics by whole-body MRI [8]. Conjugating immunomodulating drugs to these nanotracers allows their use as theranostic tools for modulating specific immune cell functions. Synthesizing therapeutic agents containing both active constituents and markers for in vivo visualization within the target organ remains a key challenge in ^19^F MRI theranostics [12]. This challenge can be addressed by utilizing iron-based nanoparticles or specific Gadolinium (Gd) or Manganese (Mn) complexes [13, 14]. A notable example of theranostics is molecular and cell therapy in the treatment of diabetes [15, 16]. In the clinical setting, confirmatory labeling and distinctions between transplanted cells and nanoparticles will ensure specific detection of therapeutic cells [17, 18]. Overall, this plenary session will underscore the transformative potential of MR theranostics and molecular imaging.

The first educational session is on multimodal imaging for theranostics and will give an update on established and emerging imaging and spectroscopic modalities in theranostics. Starting with methods in nuclear medicine, the nuclear theranostic approach aims to customize the management of various human diseases, improve patient selection, and enhance prognosis, while avoiding futile and costly diagnostic and therapeutic activities [19]. The aim is to engage a given target in dysfunctional cells or tissues. Although nuclear theranostics has primarily focused on oncology, significant novel applications are rapidly gaining traction in cardiology and neurology [20, 21]. The recent development of new radionuclide-based therapies has re-energized the field of targeted-radiotherapy [22]. Concomitantly, there is a growing recognition that theranostics can serve as convenient drug delivery systems, making theranostic strategies particularly appealing to large pharmaceutical companies seeking to develop more selective and efficient therapies [23]. The modality of MR spectroscopy (MRS) is instrumental in non-invasively elucidating tumor metabolism, particularly in adult-type diffuse gliomas, and is proving indispensable in theranostic approaches for timely diagnosis and treatment. Prognosis in adult-type gliomas pivots on mutations in isocitrate dehydrogenase (IDH) and chromosome 1p/19q codeletion [24]. Mescher–Garwood point-resolved spectroscopy (MEGA-PRESS) enables the simultaneous detection of 2–hydroxyglutarate (2HG)—a direct, downstream marker of IDH mutation [25]—and cystathionine, which accumulates preferentially in 1p/19q-codeleted gliomas [26], as demonstrated in both research and clinical settings [27]. These studies highlight the high specificity of MEGA-PRESS for both predictors in mutated gliomas, underscoring the importance of understanding the neurochemical profile for early diagnosis, compared to current standard diagnostic classifications [24]. Rapid, accurate, and noninvasive prognosis stratification of diffuse glioma with edited MRS will be essential to expedite routine workup for patients with diffuse gliomas, thereby facilitating access to IDH inhibitor treatment. Hyperpolarization methods offer an unprecedented boost in MR sensitivity via non-destructive manipulation of quantum spin state populations (typically of ^13^C & ^1^H). This provides a unique promise for in-vivo drug spatial localization [28] and metabolic probing [29]. Target nuclei can be incorporated into drug molecules as motifs or molecular tags. Hyperpolarization methods include dynamic nuclear polarization (DNP) [30], parahydrogen-induced polarization (PHIP) [31] and signal amplification by reversible exchange (SABRE) [32]. Following hyperpolarization, drug distribution and metabolism can be tracked via MRS(I) methods. The enhanced signal persists for a limited time; therefore, the implementation of cutting-edge, rapid MR methodologies and chemical manipulation for long magnetic lifetimes is imperative. Mass spectrometry adds a powerful approach for studying drug spatial localization. Additionally, MR hardware can be manipulated for targeted magnetic delivery to increase the efficacy of therapeutics [33, 34] and complement the diagnostic potential of the discussed approaches.

The second educational session will focus on invasive interventional MRI methods as theranostic tools, highlighting MRI’s versatility for needle-based therapeutic interventions [35, 36]. Thermoablation treatments including laser-induced interstitial laser therapy (LITT), radiofrequency ablation (RFA), microwave ablation (MWA), and cryoablation (CA), established for coagulating various tumors, notably in the brain, liver, kidney, and prostate. While these procedures are typically monitored using CT or ultrasound, MR imaging offers real-time temperature monitoring through MR-thermometry, enhancing therapy precision. However, challenges like breathing or residual bleeding-induced artifacts on MR-thermometry persist. Technological advances, including lower field strength MR units, aim to enhance the utility of MR-guided needle-based interventions for treatments. MRI guidance is crucial for planning treatments in vital organs like the liver, breast, and prostate, aiding in biopsy, dosimetry, and improving outcomes [37, 38]. In liver interventions, MRI even enables intraprocedural dosimetry during tumor radioembolization [39]. Leveraging interventional MRI offers distinct advantages, including real-time imaging guidance, enhanced accuracy, and improved patient outcomes. Brain surgery is another aspect that benefits interventional MRI. Intraoperative MRI–guided brain surgery enhances effective and safe tumor resection [40, 41], and the treatment of epilepsy [42]. Recent advances include MRI-guided focused ultrasound for treating tremors in parkinsonism. This innovative technique enables precise targeting and real-time thermal monitoring using MR thermometry [43]. Despite its benefits in neurosurgery, intraoperative MRI remains limited due to safety concerns. Strict safety protocols and personnel training are crucial to prevent accidents [44].

The third educational session will be non-invasive interventional MRI methods as theranostic tools. MR-guided high-intensity focused ultrasound (MR-HIFU) is an interventional treatment using HIFU that is guided by MRI for spatial treatment planning as well as monitoring of tissue heating or treatment effects. Ultrasound waves can be focused deep within a patient’s body. Within the focus region, energy dissipation leads either to heating or to a mechanical disruption of tissue and cellular structures, depending on the wave intensity and the exact pulse sequence [45–47]. HIFU uses continuous sonications to thermally ablate tissue or induce local hyperthermia [48]. Besides offering soft tissue contrast, MRI is used to monitor near-real-time temperature mapping for feedback to the HIFU transducer. This ensures a defined thermal dose for tissue ablation and constant temperatures during hyperthermia treatments. Current clinically approved MR-HIFU applications are based on thermal tissue ablation, for the treatment of uterine fibroids, desmoid tumors, or painful bone diseases. A new clinical application is histotripsy which is induced by pulsed HIFU and involves mechanical ablation of tissue without significant heat deposition [45]. For this, other MR contrast mechanisms that monitor non-thermal tissue degradation provide feedback during therapy. The concepts of MR-HIFU will be reviewed with examples of current clinical applications and ongoing trials, also focusing on the translation of preclinical work. The potential application of histotripsy in oncology will be discussed. ThermalMR combines diagnostic MRI with targeted local thermal therapy using radiofrequency (RF) applicators in an integrated system [49]. Fighting fire with fire, hyperthermia is an adjunct treatment to enhance the efficacy of other anti-cancer treatments: chemotherapy, radiotherapy and immunotherapy [50] and has clinical potential in targeted drug delivery using thermo-responsive nano-carriers [51]. ThermalMR uses RF antenna arrays to selectively increase the temperature of a target region and is governed by RF features such as the frequency and geometry of phased arrays [52, 53]. The objective is to ensure uniform magnetic transmission fields for MRI and MR thermometry and facilitate targeted control of electric fields for thermal therapy. There will be a focus on ThermalMR as it explores temperature’s role in biology and disease, introducing thermal cancer phenotyping, to advance thermal theranostics [54]. MRI-guided radiation therapy (MRgRT) represents an unprecedented therapeutic advantage compared to X-ray-based radiotherapy delivery systems by leveraging real-time imaging to customize treatments to each patient’s tumor anatomy, ensuring precise targeting while sparing radiation exposure to surrounding healthy tissues [55]. This transformative technology brings about a paradigm shift in the workflow of radiation oncology, demanding enhanced coordination among multidisciplinary teams to ensure precise treatment delivery. Upon implementation, it opens avenues for novel applications in radiation therapy, enabling the safe delivery of higher doses with enhanced preservation of healthy tissues, ultimately optimizing patient outcomes. The technical intricacies of advanced linear accelerators capable of delivering MRgRT will be outlined, along with a comprehensive summary of published experiences to date, emphasizing oncological outcomes and highlighting forthcoming challenges. MRI-guided neuromodulation is another interventional MRI that shows significant promise in neurological disorders [56, 57]. It includes techniques such as transcranial magnetic stimulation (TMS) and transcranial direct/alternate current stimulation (tDCS/tACS). Structural MRI is essential for modelling electric fields, while concurrent measurements with functional MRI (fMRI) and/or electroencephalography (EEG) are increasingly attracting more interest. Additionally, leveraging further multimodal imaging, including diffusion MRI (dMRI), holds the potential for more accurate targeting through the use of structural connectivity, based on real-time tractography [58]. Emphasis will be made on the integration of technologies as this not only refines therapeutic interventions but also deepens our understanding towards diagnostics, paving the way for more precise and personalized treatments particularly for psychiatric disorders.

The fourth educational session will be on the role of MRI in drug development and will focus on perspectives from academia and industry. Particularly in industry, positron emission tomography (PET) remains the major player during the drug discovery and development process, and during preclinical and clinical trials [59]. The use of hybrid PET-MRI enhances pharmacokinetics and pharmacodynamics, by offering simultaneous structural, microstructural, and functional information. In rare diseases, comprehensive PET-MR protocols are crucial for acquiring multimodal information on structural and functional tissue integrity. Identifying disease biomarkers and treatment responses requires careful consideration of hardware and software factors, such as radiotracer selection, MR acquisition protocols, QC procedures that ensure robust acquisitions and post-processing reproducibility to estimate disease-related and treatment-response-related metrics. Next attention will be paid to the contribution of preclinical MRI to drug development. Non-invasive imaging of whole organisms provides invaluable insights into physiology and pathology, including immune responses, surpassing what can be achieved through cell culture or organoid experiments. In vivo MRI provides dynamic, real-time data, unlike postmortem tissue analysis, which offers only a snapshot at a single time point. MRI stands out among non-invasive imaging methods due to its ability to provide a multi-parametric view, enabling the assessment of various physiological and metabolic parameters. These parameters inform on toxicity, biodistribution, efficacy, mode of action, immune response, and potential adverse effects. Despite recent advances in sensitivity and specificity, the role of preclinical MRI in drug development has evolved rather than dramatically changed in the past two decades [60, 61]. Nonetheless, preclinical MRI remains essential for adhering to the 3R principle in drug development [62]. Advancing MRI biomarkers in drug development is crucial [63, 64]. MRI can influence various stages, from preclinical to clinical trials. During the preclinical stage, MRI reveals disease mechanisms, aiding target and drug validation. Early on, it assesses pharmacokinetics and tissue distribution for safety. In clinical trials, MRI biomarkers quantify treatment efficacy and safety, expediting decision timelines when determined as early, sensitive gauges of disease progression. However, challenges persist: MRI methods must be sensitive and specific to the disease progression or treatment response; integrated with other clinical data early on to provide a more comprehensive overview [65]. Quantitative measures are essential for precise monitoring [66], requiring reproducible imaging protocols. Regulatory approval is needed for MRI as a surrogate endpoint or companion diagnostic. Finally, MRI must be cost-effective and widely accessible for widespread use.
