# Correction to: MR beyond diagnostics at the ESMRMB annual meeting: MR theranostics and intervention

**DOI:** 10.1007/s10334-024-01201-7

**Published:** 2024-09-23

**Authors:** Milan Hájek, Ulrich Flögel, Adriana A. S. Tavares, Lucia Nichelli, Aneurin Kennerley, Thomas Kahn, Jurgen J. Futterer, Aikaterini Fitsiori, Holger Grüll, Nandita Saha, Felipe Couñago, Dogu Baran Aydogan, Maria Eugenia Caligiuri, Cornelius Faber, Laura C. Bell, Patrícia Figueiredo, Joan C. Vilanova, Francesco Santini, Ralf Mekle, Sonia Waiczies

**Affiliations:** 1 Department of Diagnostic and Interventional Radiology, Institute for Clinical and Experimental Medicine, Prague, Czech Republic; 2 Experimental Cardiovascular Imaging, Institute for Molecular Cardiology, Heinrich Heine University Düsseldorf, Düsseldorf, Germany; 3 Centre for Cardiovascular Sciences and Edinburgh Imaging, University of Edinburgh, Edinburgh, UK; 4 Sorbonne Université, Inserm, CNRS, UMR S 1127, Paris Brain Institute, ICM, Paris, France; 5 Department of Neuroradiology, AP-HP, Pitié-Salpêtrière Hospital, Paris, France; 6 Department of Sports and Exercise Science, Institute of Sport, Manchester Metropolitan University, Manchester, UK; 7 Department of Biology, University of York, York, UK; 8 Department of Diagnostic and Interventional Radiology, University of Leipzig, Leipzig, Germany; 9 Minimally Invasive Image-Guided Intervention Center (MAGIC), Department of Medical Imaging, Radboudumc, Nijmegen, The Netherlands; 10 Unit of Diagnostic and Interventional Neuroradiology, Diagnostic Department, University Hospitals of Geneva, Geneva, Switzerland; 11 Institute of Diagnostic and Interventional Radiology, Faculty of Medicine, University Hospital of Cologne, University of Cologne, Cologne, Germany; 12 Department of Chemistry, Faculty of Mathematics and Natural Sciences, University of Cologne, Cologne, Germany; 13 Max-Delbrück-Centrum Für Molekulare Medizin (MDC), Berlin Ultrahigh Field Facility, Berlin, Germany; 14 Charité-Universitätsmedizin Berlin, Berlin, Germany; 15 Experimental and Clinical Research Center (ECRC), A Joint Cooperation Between the Charité Medical Faculty and the MDC, Berlin, Germany; 16 Department of Radiation Oncology, Hospital Universitario San Francisco de Asís, Hospital Universitario Vithas La Milagrosa, GenesisCare, 28010 Madrid, Spain; 17 A.I. Virtanen Institute for Molecular Sciences, University of Eastern Finland, Kuopio, Finland; 18 Neuroscience Research Center, Department of Medical and Surgical Sciences, Università Degli Studi “Magna Graecia”, Catanzaro, Italy; 19 Translational Research Imaging Center (TRIC), Clinic of Radiology, University of Münster, Münster, Germany; 20 Early Clinical Development, Genentech Inc., South San Francisco, USA; 21 Institute for Systems and Robotics, ISR-Lisboa, Lisbon, Portugal; 22 Department of Bioengineering, Instituto Superior Técnico, Universidade de Lisboa, Lisbon, Portugal; 23 Department of Radiology, Clínica Girona, Institute of Diagnostic Imaging (IDI) Girona, University of Girona, 17004 Girona, Spain; 24 Department of Radiology, University Hospital of Basel, Basel, Switzerland; 25 Basel Muscle MRI, Department of Biomedical Engineering, University of Basel, Basel, Switzerland; 26 Center for Stroke Research Berlin (CSB), Charité - Universitätsmedizin Berlin, Berlin, Germany

**Correction to: Magnetic Resonance Materials in Physics, Biology and Medicine (2024) 37:323–328** 10.1007/s10334-024-01176-5

In the original version of this article, author’s name Aikaterini Fitsiori was incorrectly written as Aikaterini Firsiori.

